# Fibroblast growth factor 2 (FGF2) regulates cytoglobin expression and activation of human hepatic stellate cells via JNK signaling

**DOI:** 10.1074/jbc.M117.793794

**Published:** 2017-09-15

**Authors:** Misako Sato-Matsubara, Tsutomu Matsubara, Atsuko Daikoku, Yoshinori Okina, Lisa Longato, Krista Rombouts, Le Thi Thanh Thuy, Jun Adachi, Takeshi Tomonaga, Kazuo Ikeda, Katsutoshi Yoshizato, Massimo Pinzani, Norifumi Kawada

**Affiliations:** From the ‡ Department of Hepatology,; § Endowed Laboratory of Synthetic Biology, and; ¶ Department of Anatomy and Regenerative Biology, Graduate School of Medicine, Osaka City University, Osaka 545-8585, Japan,; the ‖ Regenerative Medicine and Fibrosis Group, Institute for Liver and Digestive Health, University College London, Royal Free, London NW3 2PF, United Kingdom, and; the ** Laboratory of Proteome Research, Proteome Research Center, National Institute of Biomedical Innovation, Osaka 567-0085, Japan

**Keywords:** c-Jun N-terminal kinase (JNK), fibroblast growth factor (FGF), fibrosis, hepatic stellate cell (HSC), liver, cytoglobin

## Abstract

Cytoglobin (CYGB) belongs to the mammalian globin family and is exclusively expressed in hepatic stellate cells (HSCs) in the liver. In addition to its gas-binding ability, CYGB is relevant to hepatic inflammation, fibrosis, and cancer because of its anti-oxidative properties; however, the regulation of *CYGB* gene expression remains unknown. Here, we sought to identify factors that induce CYGB expression in HSCs and to clarify the molecular mechanism involved. We used the human HSC cell line HHSteC and primary human HSCs isolated from intact human liver tissues. In HHSteC cells, treatment with a culture supplement solution that included fibroblast growth factor 2 (FGF2) increased CYGB expression with concomitant and time-dependent α-smooth muscle actin (αSMA) down-regulation. We found that FGF2 is a key factor in inducing the alteration in both CYGB and αSMA expression in HHSteCs and primary HSCs and that FGF2 triggered the rapid phosphorylation of both c-Jun N-terminal kinase (JNK) and c-JUN. Both the JNK inhibitor PS600125 and transfection of c-JUN–targeting siRNA abrogated FGF2-mediated CYGB induction, and conversely, c-JUN overexpression induced CYGB and reduced αSMA expression. Chromatin immunoprecipitation analyses revealed that upon FGF2 stimulation, phospho-c-JUN bound to its consensus motif (5′-TGA(C/G)TCA), located −218 to −222 bases from the transcription initiation site in the *CYGB* promoter. Of note, in bile duct–ligated mice, FGF2 administration ameliorated liver fibrosis and significantly reduced HSC activation. In conclusion, FGF2 triggers *CYGB* gene expression and deactivation of myofibroblastic human HSCs, indicating that FGF2 has therapeutic potential for managing liver fibrosis.

## Introduction

Liver fibrosis is characterized by an excessive accumulation of extracellular matrix (ECM)^2^ components in hepatic tissue. Cirrhosis results in portal hypertension and liver failure and is associated with an increased risk of hepatocellular carcinoma (1). The discovery of new drugs targeting hepatitis viruses B and C is anticipated to dramatically decrease the number of patients with virus-related chronic liver disease (CLD) (2). In contrast, the prevalence of nonalcoholic fatty liver diseases has increased, and these are anticipated to become a leading cause of CLD (3). Regardless of the background etiologies, CLD-related liver fibrosis is a deadly disease worldwide (1); however, no Food and Drug Administration-approved anti-fibrotic drugs are currently clinically available (4).

Hepatic stellate cells (HSCs) are a dominant contributor to liver fibrosis, regardless of the underlying disease etiology (5). In the healthy liver, quiescent HSCs reside in the space of Disse between hepatocytes and sinusoidal endothelial cells (6). In response to liver injury, HSCs undergo progressive activation, transdifferentiating into collagen-producing myofibroblast-like cells and acquiring contractile properties (7, 8). During the activation process, HSCs express α-smooth muscle actin (αSMA) and synthesize fibrillar ECM, specifically type I and III collagen (COL I and III) (9). Activated HSCs also secrete growth factors and pro-fibrotic cytokines, including transforming growth factor-β1 (TGF-β1) and platelet-derived growth factor (PDGF), to stimulate HSC activation in an autocrine manner to further produce ECM (10, 11). However, experimental and clinical studies have revealed that the regression of hepatic fibrosis occurs following curative therapy of underlying liver diseases (12–14). Thus, strategies to reduce HSC activation, *i.e.* deactivation of HSCs, or to induce reversion to a quiescence-like phenotype could represent effective anti-fibrotic treatments (15, 16).

We identified a protein, originally named Stellate cell activation-associated protein (STAP), from rat cultured HSCs (17) that is currently referred to as cytoglobin (CYGB) (18). CYGB is the fourth member of the vertebrate globin superfamily, and its sequence is highly conserved among species (18). CYGB has characteristic properties of a heme protein and exhibits peroxidase activity that catalyzes hydrogen peroxides and lipid hydroperoxides (17, 19). CYGB is ubiquitously expressed in all organs other than the human liver, where it is expressed solely in HSCs, and its expression is reduced in the livers of patients with CLD (20, 21). Recently, our laboratory and others have reported that CYGB plays a protective role both in neuronal cells and in the liver by reducing reactive oxygen species (ROS) (22, 23). Furthermore, the administration of human recombinant CYGB was reported to attenuate thioacetamide-induced liver fibrosis in a rat model (24). However, CYGB expression in human HSCs and its regulatory mechanism remain largely unstudied.

Here, we show, for the first time, that fibroblast growth factor 2 (FGF2) is a strong inducer of CYGB in human HSCs via the activation of c-JUN-terminal kinase (JNK)/c-JUN signaling. Moreover, FGF2 suppresses αSMA expression via the ERK-signaling pathway. We also show that FGF2 administration ameliorates liver fibrosis induced by bile duct ligation (BDL) in mice. Taken together, our study reveals the previously unrecognized FGF2-dependent induction of *CYGB* gene expression, which is accompanied by the deactivation of human HSCs and represents a novel strategy for anti-fibrotic therapy.

## Results

### Induction of CYGB expression in human hepatic stellate cell lines

**Figure 1.**
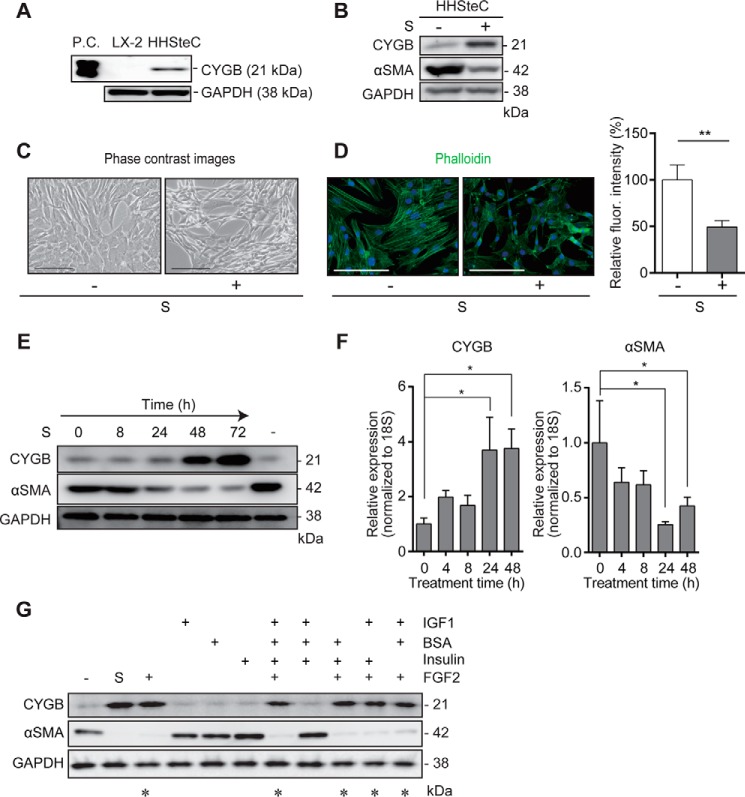
**Effect of supplement solution on CYGB and αSMA expression in HHSteCs.** *A,* expression of CYGB in LX-2 cells and HHSteCs. LX-2 cells and HHSteCs at passage 5 were cultured in DMEM with 2% FBS and SteCM with 2% FBS and supplement solution, respectively, for 72 h and were collected as precipitates consisting of 10^6^ cells for Western blotting. CYGB was expressed only in HHSteCs. GAPDH was used as a loading control. Recombinant human CYGB protein was used as a positive control (*P.C.*). *B,* levels of CYGB and αSMA proteins in HHSteCs stimulated by supplement solution for 72 h. *C,* phase-contrast images of HHSteCs cultured with or without supplement solution (*S*; 1×) for 72 h. *Scale bars,* 100 μm. *D,* F-actin expression visualized with Alexa Fluor 488-conjugated phalloidin using a fluorescence microscope. *Scale bars,* 100 μm. The relative fluorescence intensities of cellular F-actin were quantified in HHSteCs with or without supplement solution. The data represent the mean of four replicates ± S.D. **, *p* < 0.01 compared with untreated control (unpaired *t* test). *E,* time-dependent expression of CYGB and αSMA proteins in HHSteCs stimulated with supplement solution (1×). The expression was compared with the untreated control (*last lane*; −). *F,* effect of supplement solution on *CYGB* and α*SMA* mRNA expression in HHSteCs. The data are expressed as the mean ± S.D. from three independent experiments. *, *p* < 0.05 compared with 0 h (one-way ANOVA). *G,* HHSteCs were treated with combinations of the indicated factors for 72 h and then subjected to Western blot analysis for CYGB and αSMA expression. The final concentrations of each substance were as follows: IGF-1 (4 ng/ml); bovine serum albumin (10 μg/ml); insulin (7.5 μg/ml); and FGF2 (4 ng/ml). * indicates the combination(s) that induce CYGB and down-regulate αSMA proteins compared with the untreated control based on band densities measured using ImageJ software.

In our first set of experiments, CYGB expression was compared between LX-2 cells, which have been widely used and are extensively characterized as a human HSC line (25), and the human HSC line HHSteCs. HHSteCs were established and distributed by ScienCell Research Laboratories and have been used as primary human HSCs (26, 27). LX-2 cells were cultured in DMEM with 2% FBS. HHSteCs were maintained in SteCM with 2% FBS and associated supplement solution (1×). We confirmed that HHSteCs are not an immortalized cell line but are human normal diploid HSCs because they become senescent after 15 population doublings under the recommended culture conditions. As shown in Fig. 1*A*, CYGB was expressed in HHSteCs, but not in LX-2 cells, at the protein level. We noted that there was hyper-methylation of the *CYGB* promoter region in LX-2 cells but not in HHSteCs, an observation that may explain the absence of CYGB in LX-2 cells (data not shown). Supplement solution increased the CYGB protein level and conversely down-regulated the protein level of αSMA, a well-established myofibroblast and HSC activation marker, in HHSteCs (Fig. 1*B*). Along with the protein alterations, HHSteCs appeared flattened and polygonal in shape with thick bundles of stress fibers in the absence of supplement solution, whereas they exhibited a clear boundary with a thinner cell body and dissolved stress fibers in the presence of supplement solution (Fig. 1*C*). The fluorescence intensity of cellular F-actin was significantly decreased (∼50%) in supplement solution-treated HHSteCs compared with that in untreated control cells (Fig. 1*D*). HHSteCs retained a high level of CYGB expression during culture passages (data not shown) and exhibited expression profiles of well-characterized HSC-associated genes, such as desmin, neurotrophin-3, retinol-binding protein-1, and lecithin-retinol acyltransferase (supplemental Fig. 1*A*). LX-2 cells exhibited relatively low expression levels of desmin and retinol-binding protein-1 compared with HHSteCs. Thus, HHSteCs were employed for further analyses in this study.

The CYGB protein level was markedly increased by ∼560% and the αSMA protein level was decreased to ∼8.5% of the basal values, and the corresponding mRNA levels were up- and down-regulated, respectively, by supplement solution (Fig. 1, *E* and *F*). According to these observations, we speculated that supplement solution might contain substances with the potential to induce the expression of CYGB and inhibit HSC activation. To test our hypothesis, supplement solution was subjected to LC-MS/MS analysis. As a result, supplement solution was found to contain peptide components, such as human FGF2 (supplemental Fig. 2*A*; a chromatograph of FGF2 peaks), human insulin, and albumin (from bovine serum; BSA). Human insulin-like growth factor-1 (IGF-1), which has 48% amino acid sequence identity with pro-insulin, was also considered a candidate. To determine the key factors for CYGB induction in HHSteCs, the cells were exposed to the basal medium of SteCM/FBS either alone or in combination with added FGF2 (4 ng/ml), IGF-1 (2 ng/ml), BSA (10 μg/ml), or insulin (7.5 μg/ml). Immunoblot analysis revealed that the combination treatment of FGF2, IGF-1, and BSA most faithfully recapitulated the effect of supplement solution on HHSteCs (CYGB >2-fold increase and αSMA <0.5-fold decrease) as measured by the normalized band intensity (Fig. 1*G*). In addition, FGF2 alone could recapitulate the supplement solution effect in contrast to IGF-1 and BSA, each of which alone was unable to promote such an effect. Thus, we concluded that FGF2 is the major ingredient in supplement solution that induces the “supplement solution effect” on HHSteCs.

### Effects of FGF2 on the expression of CYGB and αSMA in HHSteCs

**Figure 2.**
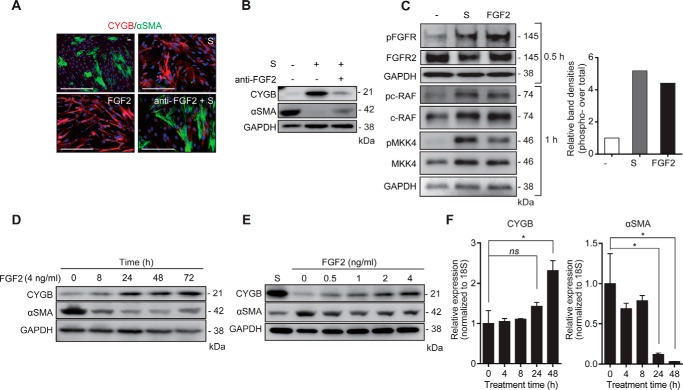
**Effect of FGF2 on CYGB and αSMA expression in HHSteCs.** *A,* dual immunofluorescence staining of CYGB (*red*), αSMA (*green*), and DAPI nuclear counterstain (*blue*) in HHSteCs following treatment with supplement solution (1×), FGF2 (4 ng/ml), and supplement solution preincubated with anti-FGF2-neutralizing antibody (2 μg/ml) for 2 h. Note that both supplement solution and FGF2 markedly enhanced CYGB and suppressed αSMA expression. The effect of supplement solution treatment was reversed by anti-FGF2 antibody treatment in HHSteCs. *Scale bars,* 100 μm. *B,* effect of preincubation of supplement solution with anti-FGF2 antibody on CYGB and αSMA protein expression in HHSteCs. GAPDH was used as a loading control. *C,* phosphorylation of FGFR (Tyr-653/654), c-RAF, and MKK4 and total FGFR2. c-RAF and MKK4 were analyzed by Western blotting. HHSteCs were stimulated with supplement solution (1×) and 4 ng/ml FGF2 at the indicated time points. GAPDH was used as a loading control. *Right inset,* the relative band density of phospho-FGFR normalized to total FGFR2 compared with the control. *D* and *E,* Western blottings of CYGB and αSMA from HHSteCs stimulated with 4 ng/ml FGF2 at the indicated time points (*D*) and with the indicated concentrations of FGF2 for 48 h (*E*). *F,* time-dependent expression of *CYGB* and α*SMA* mRNA in HHSteCs stimulated with 4 ng/ml FGF2. The data are expressed as the mean ± S.D. from three independent experiments. *ns*, not significant; *, *p* < 0.05 compared with 0 h (one-way ANOVA).

Immunocytochemical analyses revealed that recombinant human FGF2 (4 ng/ml) induced the *de novo* induction of CYGB and reduction of αSMA in a manner similar to the effect of supplement solution in HHSteCs. Furthermore, a neutralizing human FGF2 antibody (2 μg/ml) counteracted the effect of supplement solution on CYGB and αSMA expression according to both immunostaining and Western blot analysis (Fig. 2, *A* and *B*). The anti-FGF2 antibody also reversed the supplement solution-induced morphological changes and antagonized the supplement solution-regulated expression of both CYGB and αSMA proteins in HHSteCs in a dose-dependent manner (supplemental Fig. 3, *A* and *B*).

To examine the level of total FGF receptors (FGFRs) in HHSteCs, droplet digital PCR analysis was performed to assess FGFR1, FGFR2, FGFR3, and FGFR4 cDNA copy numbers in 3-day cultured HHSteCs with or without FGF2 (4 ng/ml). Although the absolute copy number of FGFR1 was the most abundant among these, only the FGFR2 copy number was significantly amplified by FGF2 treatment in HHSteCs (supplemental Fig. 3*C*). Next, we examined the level of FGFR2 protein and phospho-FGF receptor (p-FGFR; Tyr-653/654) in HHSteCs. Immunoblot experiments showed that although the level of total FGFR2 protein was unaffected, the level of p-FGFR was significantly increased in HHSteCs treated with supplement solution or FGF2 (4 ng/ml) compared with the untreated control; the ratio of p-FGFR/FGFR2 was markedly increased by 5.2- and 4.4-fold in HHSteCs treated with supplement solution and FGF2, respectively. Phosphorylation of c-RAF and MKK4, downstream signals of FGFR, was also observed (Fig. 2*C*). In addition, FGF2 produced a time- and dose-dependent induction of CYGB and a reduction of αSMA protein (Fig. 2, *D* and *E*). For example, in HHSteCs treated with 4 ng/ml FGF2, the CYGB and αSMA protein levels were up- and down-regulated by 890 and to 10%, respectively, at 72 h. Furthermore, the time dependence of these effects was confirmed at the mRNA level, although the alterations of *CYGB* and α*SMA* mRNA expression were unexpectedly significantly prolonged at 48 h (Fig. 2*F*). Both supplement solution and FGF2 also hampered the spontaneous induction of *COLIA1* mRNA expression (*i.e.* untreated control) in a time-dependent manner (supplemental Fig. 3*D*).

### FGF2 initiates CYGB transcription via the JNK pathway

**Figure 3.**
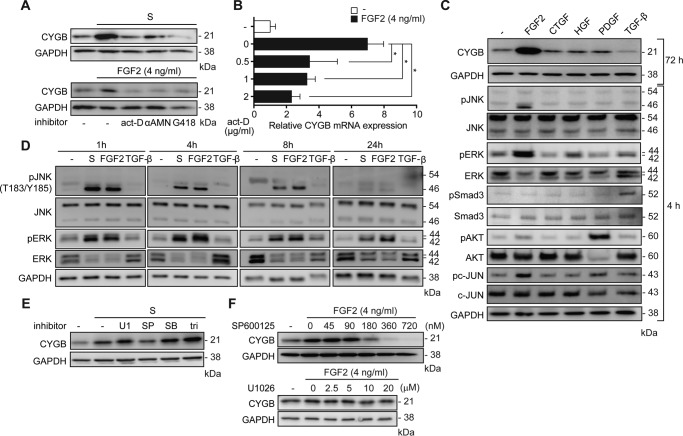
**Regulatory signaling of CYGB and αSMA expression in HHSteCs upon FGF2 and supplement solution stimulation.** *A,* effect of transcription inhibitors actinomycin D (*act-D*; 2 μg/ml) and α-amanitin (α*AMN*; 2 μg/ml), as well as a translation inhibitor, G418 (2 μg/ml), on CYGB expression under supplement solution (*upper*) and 4 ng/ml FGF2 (*lower*) stimulation in HHSteCs. *B,* effect of actinomycin D on *CYGB* mRNA expression in HHSteCs stimulated with FGF2 (4 ng/ml). HHSteCs were incubated with the indicated concentrations of actinomycin D for 72 h. *, *p* < 0.05 compared with 0-h (one-way ANOVA). *C,* expressions of CYGB and total and phospho-JNK, ERK, Smad2/3, AKT, and c-JUN in HHSteCs were assayed by Western blot analysis after treatment with medium (−) or FGF2 (4 ng/ml), CTGF (80 ng/ml), HGF (20 ng/ml), PDGF (20 ng/ml), and TGF-β1 (5 ng/ml) at the indicated time points. GAPDH was used as a loading control. *D,* HHSteCs were treated with supplement solution (1×), FGF2 (4 ng/ml), and TGF-β1 (5 ng/ml) for the indicated lengths of time (1, 4, 8, and 24 h). Total levels of phosphorylated JNK (*pJNK, T183/Y185*), JNK, phosphorylated ERK (*pERK*), and ERK were assessed using Western blot analysis. *E,* HHSteCs were treated with an MEK inhibitor, U1026 (20 μm), a JNK inhibitor, SP600125 (180 nm), a p38 inhibitor, SB203580 (64 nm), and an AKT inhibitor, triciribine (260 nm) 2 h prior to the addition of supplement solution (1×). Cell lysates were analyzed by Western blotting with antibodies against CYGB. *F,* HHSteCs were pretreated with a JNK inhibitor and an MEK inhibitor at the indicated doses prior to FGF2 (4 ng/ml) treatment. CYGB protein expression disappeared at a high dose of the JNK inhibitor SP600125 (*left*) but was unaltered by the MEK inhibitor U1026 (*right*) in the presence of FGF2 treatment.

To clarify whether supplement solution and FGF2 regulated CYGB protein expression at the transcriptional or translational level, HHSteCs were treated with FGF2 or supplement solution in the presence of actinomycin D or α-amanitin, transcriptional inhibitors, or G418, a translational inhibitor, compared with DMSO, a vehicle control. All inhibitors decreased the elevation of CYGB expression upon FGF2 (4 ng/ml) or supplement solution treatment, which confirmed that CYGB expression was regulated at both the transcriptional and translational levels (Fig. 3*A*). Furthermore, actinomycin D significantly attenuated FGF2-induced *CYGB* mRNA up-regulation in a dose-dependent manner in HHSteCs (Fig. 3*B*).

Based on the results of dose-response studies for each growth factor to optimize their concentrations (supplemental Fig. 4*A*), HHSteCs were exposed to FGF2 (4 ng/ml), CTGF (80 ng/ml), HGF (20 ng/ml), PDGF (20 ng/ml), and TGF-β1 (5 ng/ml) for 72 h. The CYGB protein level was only increased with FGF2 stimulation. Regarding intracellular signaling pathways, FGF2 triggered the phosphorylation of JNK, ERK, and c-JUN, whereas HGF, PDGF, and TGF-β induced the phosphorylation of ERK, AKT, and Smad3, respectively, 4 h after the stimulation of each growth factor (Fig. 3*C*). FGF2 and supplement solution, but not TGF-β1 (5 ng/ml) treatment, stimulated the phosphorylation of JNK and ERK; the phosphorylation of JNK peaked at 1 h and decreased after 8 h and that of ERK peaked at 4 h and continued for 24 h (Fig. 3*D*). To evaluate the pathway required for CYGB induction by FGF2, the effects of inhibitors against ERK1/2, JNK, p38, and AKT on the expression of CYGB were tested. Supplement solution-dependent CYGB induction was attenuated by SP600125 (180 nm), a JNK inhibitor, but not by U1026 (20 μm), an MEK inhibitor, triciribine (260 nm), an AKT inhibitor, or SB203580 (64 nm), a p38 inhibitor (Fig. 3*E*). In addition, upon treatment with 4 ng/ml FGF2, SP600125 induced the reduction of CYGB expression but U1026 failed to affect the CYGB protein level (Fig. 3*F*). Taken together, the FGF2–FGFR2–JNK–c-JUN pathway was elucidated as a primary pathway in the induction of CYGB in HHSteCs.

### FGF2 treatment recruits c-JUN to the proximal region of the CYGB promoter

**Figure 4.**
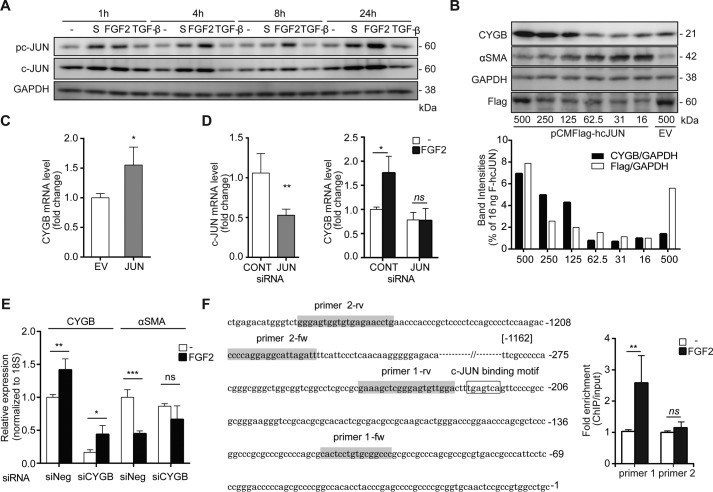
**Activation of CYGB gene transcription by FGF2 via c-JUN in HHSteCs.** *A,* HHSteCs were stimulated with supplement solution (1×), FGF2 (4 ng/ml), and TGF-β1 (5 ng/ml) for the indicated times (1, 4, 8, and 24 h). Levels of phosphorylated c-JUN (*pc-JUN*) and total c-JUN were examined using Western blotting. *B,* HHSteCs were transfected with an increasing amount of pCMFLAG-hcJUN (16, 31, 62.5, 125, 250, and 500 ng/ml). An empty vector (*EV*, 500 ng/ml) was transfected as a control. Representative relative band densities from c-JUN overexpression of CYGB, αSMA, and FLAG proteins are shown in the *bar graphs*. Band densities of CYGB and FLAG were normalized to GAPDH and compared with those of HHSteCs transfected with pCMFLAG-hcJUN (16 ng/ml) and were set to 1. *C,* significant increase in *CYGB* mRNA expression was observed in pCMFLAG-hcJUN-transfected HHSteCs (250 ng/ml) compared with those transfected with empty vector (250 ng/ml) *, *p* < 0.05 (unpaired *t* test). *D,* HHSteCs were transiently transfected with siRNA against c-*JUN*. The decreased c-*JUN* mRNA level was confirmed after transfection with the specific c-*JUN* siRNA compared with a random oligonucleotide (negative control). **, *p* < 0.01 (unpaired *t* test) (*left*). Levels of *CYGB* mRNA were examined following FGF2 (4 ng/ml) treatment for 24 h. The data are expressed as the mean ± S.D. from two independent experiments performed in triplicate. *ns*, not significant; *, *p* < 0.05 compared with 0 h (one-way ANOVA) (*right*). *CONT,* control. *E,* HHSteCs were transiently transfected with siRNA against human *CYGB*. siRNA-transfected HHSteCs were treated with and without FGF2 (4 ng/ml) for 48 h. The decreased *CYGB* mRNA level was confirmed after transfection with *CYGB* siRNA compared with a random oligonucleotide (negative (*Neg*) control). The level of α*SMA* mRNA was investigated in siRNA-transfected HHSteCs with the treatment of FGF2. *ns*, not significant; *, *p* < 0.05 compared with the untreated control (unpaired *t* test); **, *p* < 0.01, and ***, *p* < 0.001. *F,* ChIP analysis of phospho-c-JUN at the *CYGB* promoter was analyzed by quantitative RT-PCR. Primers were designed at the c-JUN-binding motif (primer 1; containing the TGA(C/G)TCA DNA sequence) and non-c-JUN-binding region in the *CYGB* promoter within 1500 bp upstream of the transcriptional initiation site in HHSteCs treated with or without FGF2 (4 ng/ml) for 6 h. The untreated control was set as 1, and the result was presented as relative fold enrichment. The data are expressed as the mean ± S.D. from three independent studies. *ns*, not significant; **, *p* < 0.01 compared with the untreated control (unpaired *t* test with Welch's correction).

An increase in phosphorylated c-JUN and total c-JUN was observed upon treatment with FGF2 (4 ng/ml) and supplement solution, but not with TGF-β1 (5 ng/ml), in HHSteCs as early as 1 h after exposure (Fig. 4*A*). Next, the role of c-JUN in the expression of CYGB was investigated by transfecting 15.6–500 ng of a pCMFLAG-hcJUN vector into HHSteCs for 72 h. The c-JUN protein level, as determined by FLAG immunoblotting, was markedly increased by 125–250 ng of pCMFLAG-hcJUN vector transfection, resulting in a significant induction of CYGB protein expression by more than 7-fold (Fig. 4*B*). Interestingly, αSMA expression was reduced markedly at a high level of *de novo* CYGB protein in a specular manner. The induction of c-JUN led to *CYGB* mRNA expression, as shown in Fig. 4*C*. In contrast, transfection of siRNA directed against c-*JUN* significantly decreased the FGF2-induced *CYGB* mRNA expression (Fig. 4*D*). Additionally, the inhibitory effect of FGF2 on the activation of human HSCs was examined by using an siRNA system targeted to human *CYGB* (siCYGB). The knockdown of *CYGB* mRNA by siCYGB blunted the FGF2-triggered down-regulation of α*SMA* mRNA in HHSteCs. This result demonstrates the direct involvement of human CYGB in the inhibitory effect of FGF2 on human HSC activation (Fig. 4*E*).

Furthermore, we performed ChIP assays followed by quantitative RT-PCR to evaluate the direct binding of c-JUN to the *CYGB* promoter region in HHSteCs after 6 h of exposure to FGF2 (4 ng/ml). The primers were designed by proximity to the binding of the c-JUN consensus motif (5′-TGA(C/G)TCA), which is located −218 to −222 bases from the transcription initiation site in the *CYGB* promoter. ChIP-quantitative PCR analyses showed that c-JUN associated with chromatin at distinct sites, with 2.6-fold enrichment in binding to the anti-phospho-c-JUN antibody *versus* the untreated control (primer 1). There was no significant difference in ChIP-quantitative PCR analysis with the anti-phospho-c-JUN antibody of the control sites (primer 2; lacking a c-JUN motif) in the *CYGB* promoter compared with the untreated control (Fig. 4*F*).

### FGF2 and supplement solution induce a quiescence-like phenotype in HHSteCs and human primary-cultured HSCs

**Figure 5.**
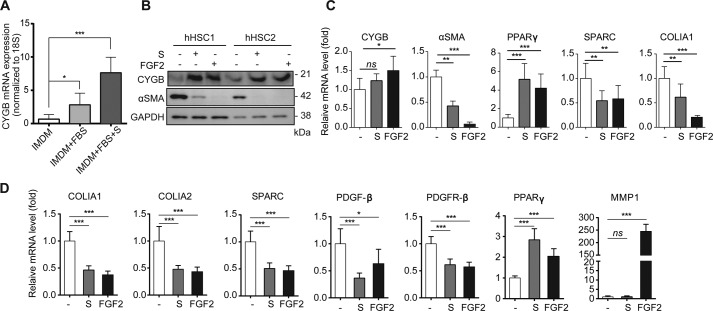
**FGF2 enhances CYGB and suppresses αSMA expression in human primary HSCs.** *A,* human HSCs were cultured under different conditions, *i.e.* IMDM only, IMDM, 2% FBS, and IMDM, 2% FBS with supplement solution at the 5th passage. The level of *CYGB* was assessed by quantitative RT-PCR. *, *p* < 0.05; ***, *p* < 0.005; compared with the IMDM control (*n* = 3, one-way ANOVA). *B,* effect of supplement solution and FGF2 on the CYGB and αSMA expression levels of two individual primary human HSC (hHSC1 and hHSC2) preparations. *C,* relative mRNA expression of *CYGB*, α*SMA, PPAR*γ, *SPARC*, and *COLIA1* was examined following supplement solution (1×) and FGF2 (4 ng/ml) treatment for 5 days in primary hHSCs. The data are expressed as the mean ± S.D. from two independent experiments performed in triplicate. *, *p* < 0.05; **, *p* < 0.001l; ***, *p* < 0.005, *ns*, not significant, compared with the untreated control (unpaired *t* test with Welch's correction). *D,* expression profiles of genes that are generally up-regulated in activated HSCs: *COLIA1, COLIA2, SAPRC, PDGF*-β, and *PDGFR*-β in quiescent HSCs; *PPAR*γ and *MMP-1* in HHSteCs. mRNA expression was assessed after 72 h of treatment with supplement solution (1×) and FGF2 (4 ng/ml) using quantitative RT-PCR. Untreated cells were used as representative controls. The data are expressed as the mean ± S.D. from two independent experiments performed in triplicate. *p* < 0.05 (*n* = 3, unpaired *t* test with Welch's correction).

Next, we assessed whether FGF2 alters CYGB and αSMA expression in primary-cultured hHSCs. Primary hHSCs were cultured in either SteCM complete medium or 2% FBS Iscove's modified DMEM (IMDM). Primary hHSCs maintained relatively high *CYGB* expression during the cultivation in IMDM with supplement solution after the 5th passage (Fig. 5*A*). In SteCM-cultured hHSCs without supplement solution, FGF2 (4 ng/ml) increased CYGB and reduced αSMA at the protein level (results from two of four HSC preparations are shown in Fig. 5*B*), similar to the effect of supplement solution. The relative mRNA expression of *CYGB* and α*SMA* was also increased and reduced by FGF2, respectively, compared with nontreated hHSCs. We also examined the effect of supplement solution and FGF2 on the expression of PPARγ, which is a master regulator of adipogenesis and is reported to be suppressed in cultured–activated human and rat HSCs (28, 29). Supplement solution and FGF2 significantly increased the relative mRNA expression of PPARγ in hHSCs. Nakatani *et al.* (30) demonstrated that secreted protein acidic and rich in cysteine (SPARC) was co-expressed in PDGF/αSMA-positive HSCs in human liver specimens and was significantly increased during human chronic hepatitis. We found that the mRNA levels of SPARC and COLIA1 were significantly reduced by supplement solution and FGF2 treatment in hHSCs (Fig. 5*C*).

Additionally, we evaluated the effect of FGF2 on well-known up-regulated genes in both activated HSCs, such as *COLIA1, COLIA2, SPARC, PDGF*-β, and the PDGFR-β receptor *PDGFR*-β, and down-regulated genes, such as *PPAR*γ and matrix metalloproteinase-1 (*MMP-1*), using HHSteCs. FGF2 significantly decreased the mRNA levels of *COLIA1, COLIA2, SPARC, PDGF*-β, and *PDGFR*-β but significantly increased the *PPAR*γ and **MMP-1** mRNA levels in HHSteCs (Fig. 5*D*). Taken together, FGF2 triggers the induction of a quiescence-like phenotype in human HSCs.

### FGF2 treatment attenuates BDL-induced liver fibrosis in mice

**Figure 6.**
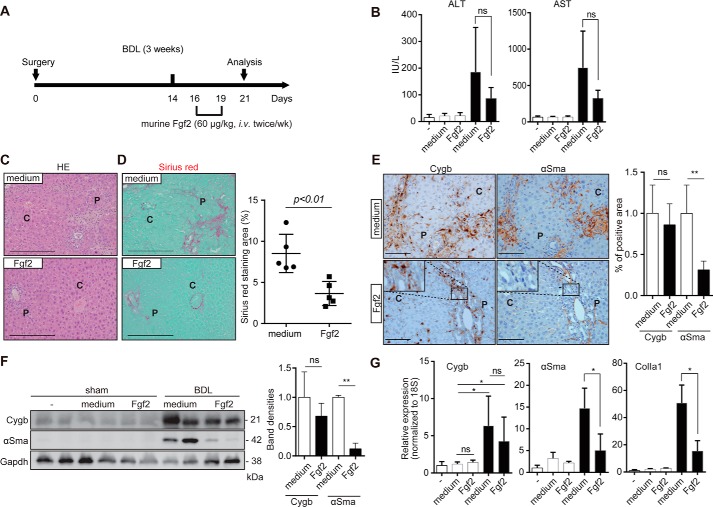
**Fgf2 ameliorates liver fibrosis in a mouse BDL model.** *A,* schematic illustration of the BDL-induced liver fibrosis model. Two weeks after surgery, mice were intravenously injected twice a week with IMDM (medium, control vehicle) and 60 μg/kg recombinant mouse Fgf2 in IMDM, and then were euthanized at day 21 for further analyses. *B,* serum levels of ALT and AST of sham-operated mice (−; *n* = 3), sham-operated and medium (IMDM)-injected mice (*n* = 3), sham-operated and IMDM/Fgf2-injected mice (*n* = 3), BDL-operated and IMDM-injected mice (*n* = 6), and BDL-operated and IMDM/Fgf2-treated mice (*n* = 6) were examined. The *open columns* and *closed columns* indicate sham-operated and bile duct–ligated mice, respectively. *ns*, not significant. *C* and *D,* liver fibrosis was evaluated by H&E (*HE*) staining (*C*) and Sirius red staining (*D*) in liver sections of BDL medium (*medium*) and BDL-Fgf2 (*Fgf2*)–treated mice. *P,* portal triad; *C,* central vein, *Scale bars,* 200 μm. The *graphs* show the quantification of Sirius red-stained and αSMA-positive areas in the control BDL medium group *versus* the BDL-Fgf2–treated group. Note the significant reduction of collagen deposition with Fgf2 treatment. The results are shown as the median ± S.E. for biological replicates (*n* = 6). Student's *t* test, *p* < 0.01. *E,* immunohistochemistry for Cygb (*right*) and αSma (*left*) in serial liver sections from BDL-operated mice with medium (*upper*) and Fgf2 (*lower*) injections. *Magnified views* of the enclosed area show that cells stained positive for Cygb but negative for αSma. *Scale bar*, 100 μm (×20 magnification). The percentage areas of Cygb and αSma positivity were measured in three random fields at ×10 magnification. The data are presented as Mann-Whitney *U* test analyses as the median ± S.E. *ns*, not significant; **, *p* < 0.01 between BDL-Fgf2–treated mice and BDL medium–treated mice. *F,* representative bands from Western blot analysis of Cygb and αSma expression in sham- and BDL-operated mice with both control (*medium*) and Fgf2 injections. CYGB expression was increased with both medium and Fgf2 treatment, whereas αSma expression was significantly reduced in BDL-operated Fgf2-injected mice. The relative band densities of Cygb and αSma compared with the BDL-operated control for at least three different animals from each group were examined. Gapdh served as a loading control (*n* = 3 in each group). The data are presented as the mean ± S.E. for biological replicates (*n* = 3 for each group). Unpaired *t* test, *ns*, not significant; **, *p* < 0.01. *G,* relative mRNA levels of *Cygb*, α*Sma*, and *ColIa1* in liver tissues of sham-operated and BDL mice injected with medium and Fgf2 were analyzed by quantitative RT-PCR. The *open columns* and *closed columns* indicate sham-operated and bile duct–ligated mice, respectively. The data are presented as one-way ANOVA as the median ± S.E. *, *p* < 0.05.

Based on the effect of FGF2 on the CYGB expression and activation status of cultured primary hHSCs and HHSteCs, we next assessed the potential role of exogenous FGF2 on liver fibrosis. To this end, mice were subjected to BDL-induced liver fibrosis and were administered recombinant mouse Fgf2 (60 μg/kg) via the tail vein twice per week following 2 weeks of BDL (Fig. 6*A*). The serum levels of aspartate aminotransferase (ALT) and alanine transaminase (AST) tended to decrease (albeit not significantly) with Fgf2 treatment (Fig. 6*B*). H&E and Sirius red staining revealed hepatocyte damage with bile duct hyperplasia and extended fibrosis predominantly around the portal vein areas of the BDL-control livers, whereas Fgf2 treatment markedly attenuated these manifestations (Fig. 6, *C* and *D*). The quantitative Sirius red-positive areas were significantly decreased in Fgf2-treated mouse livers compared with control treatment (Fig. 6*D*). Both Cygb- and αSma-positive cells were propagated around portal vein areas in medium-injected BDL murine livers. Fgf2 administration maintained Cygb expression but markedly suppressed αSma expression around portal vein areas (Fig. 6*E*). Immunoblot analysis confirmed the maintenance of Cygb and marked reduction of αSma by Fgf2 administration, compared with control medium injection, in BDL-treated murine livers (Fig. 6*F*). The levels of *Cygb* mRNA were increased in BDL murine livers compared with those of the controls, but there were no differences between the medium and FGF2-injected BDL groups. However, the expression levels of α*Sma* and *ColIa1* mRNA were significantly reduced in FGF2-treated mouse livers compared with those in the medium-treated mouse livers (Fig. 6*G*). These observations suggest that the administration of Fgf2 attenuated BDL-induced liver fibrosis, in part, by reducing the number of activated HSCs.

## Discussion

We have demonstrated that FGF2 is a key modulator of an activated phenotype of human HSCs by up-regulating CYGB expression via the JNK/c-JUN pathway. Thus, we have delineated previously unrecognized and temporally controlled cascades that differentially regulate CYGB and activate HSC-related genes, such as α*SMA, COL1A1, COL1A2*, and *SPARC*, in response to FGF2 in human HSCs. We have also revealed that pharmacological application of FGF2 attenuates the progression of liver fibrosis in an experimental murine BDL model, indicating that FGF2 is a candidate anti-fibrotic agent for the treatment of liver fibrosis.

During liver injury, HSCs undergo sequential events of activation, including cell proliferation, contractility, matrix degradation, retinoid loss, and cytokine and chemokine release (31), leading to fibrosis development and the deposition of ECM rich in type I collagen. During this process, HSCs are exposed to fibrogenic factors, such as TGF-β1, interleukin-1 (IL-1), IL-6, PDGF, tumor necrosis factor-α, and ROS (32), which are derived from injured hepatocytes, activated endothelial cells, and Kupffer cells (31, 33). The overproduction of ROS leads to the depletion of anti-oxidants in the injured liver, and activated HSCs become more susceptible to oxidative stress because of their inability to detoxify lipid peroxidation products via reduced detoxification enzymes, such as glutathione *S*-transferases and catalase (34). Thus, the imbalance between the generation of ROS and the anti-oxidant defense system of cells is causatively deleterious to the cells. Overall, inducing endogenous CYGB without harming other biological events may be advantageous when blocking HSC activation during liver fibrosis.

In this study, we investigated the role of FGF2 in the transcriptional regulation of CYGB in human HSCs. FGFs are a mediator of fibroblast growth and are widely expressed in various cell types (35). FGF2 has been considered to be pro-fibrotic because of its potential chemotactic and mitogenic activities in HSCs in culture and the observed delay in excisional skin wound healing in mice lacking FGF2 (36–38). In contrast, accumulating evidence has suggested that FGF2 is a potent anti-fibrogenic factor. FGF2 was not required for the generation of bleomycin-induced lung fibrosis, whereas it was essential for lung epithelial recovery (39). FGF2 was reported to be one of the key factors in promoting the inactivation of HSCs to a more quiescent-like phenotype *in vitro* (40). Finally, Pan *et al.* (41) showed an opposing function between low- and high-molecular-weight FGF2, and the administration of low-molecular-weight FGF2 effectively reversed liver fibrosis. In addition to the previous study results obtained using rodent models and cells, we revealed here that FGF2 is a key molecule for CYGB induction and for maintaining human HSCs in a quiescent-like phenotype.

This study demonstrated that a signaling network orchestrates the initiation of JNK and c-JUN phosphorylation, resulting in the alteration of CYGB expression in HHSteCs upon FGF2 stimulation. We also discovered direct binding of c-JUN at the *CYGB* promoter in proximity to the c-JUN-binding site upon FGF2 treatment. In human HSCs, our observation suggests that the fine-tuning of FGF2-mediated CYGB expression is tightly regulated and further deactivates HSCs, as demonstrated by a marked reduction in αSMA expression, a well-known marker of HSC activation. Previous work from our group showed that the transient knockdown of *Cygb* by siRNA results in increased αSma expression in primary mouse HSCs (22) and that αSma expression was notably high in HSCs isolated from CYGB knock-out mice (42), implying that CYGB associates with αSMA expression during the activation of HSCs. Moreover, we observed that overexpression of c-JUN markedly up-regulated CYGB and down-regulated α*SMA* expression in HHSteCs (Fig. 4*B*) and that knockdown of human *CYGB* by siCYGB counteracted FGF2-triggered down-regulation of αSMA mRNA in HHSteCs (Fig. 4*E*). Taken together, these observations indicate the involvement of human CYGB in the inhibitory effect of FGF2 on human HSC activation, although the detailed molecular mechanism of this phenomenon needs to be studied further.

Although the administration of *Fgf2 in vivo* to BDL mice was insufficient to induce Cygb expression in the liver (Fig. 6*F*), this result could be explained by masking of the pharmacological action of exogenous murine Fgf2 via the spontaneous and robust induction of Cygb, which is likely to attenuate murine HSC activation, a process initiated by the still unidentified molecular mechanisms (5, 8), and to increase the Cygb-positive HSC numbers in BDL murine livers. It should be noted that Fgf2 administration maintained Cygb expression around portal vein areas rich in myofibroblastic HSCs at day 21 but markedly suppressed αSma expression (Fig. 6*E*), indicating the contribution of Fgf2 to sustained expression of Cygb. Our previous and ongoing studies clearly demonstrated that BDL-induced liver fibrosis was markedly enhanced in Cygb-deficient mice (43) and conversely limited in conditional CYGB-transgenic mice,^3^ demonstrating the anti-fibrotic role of Cygb in the murine liver. It should also be noted that exogenously administered FGF2 may target other hepatic cells for its anti-fibrotic effects; Pan *et al.* (41) demonstrated that the administration of recombinant low-molecular-weight FGF2 markedly reduced CCl_4_-induced liver fibrosis via the suppression of delta-like 1 (Dlk-1) expression in damaged hepatocytes, resulting in a decreased level of Dlk-1 protein in the liver and serum to prevent HSC activation. To understand human pathological conditions, Fgf2-mediated regulation of HSC activation via Cygb *in vivo* needs to be further validated using various animal models.

In summary, these data revealed that FGF2 is a novel key inducer of CYGB and a suppressor of αSMA, COLIA1, and COLIA2 in human HSCs. Together with our *in vivo* results, we hypothesize that FGF2 is one of the master regulators of HSC activation. The identification of critical regulatory pathways to control the activation of myofibroblasts, including HSCs, is profoundly important for developing a therapeutic strategy for organ fibrosis. Our molecularly based study of the effect of FGF2 on human HSCs has led to greater understanding of the pathogenesis of hepatic fibrogenesis and provided a strategy to promote the resolution of liver fibrosis.

## Experimental procedures

### Cell culture

Human hepatic stellate cells, referred to as HHSteCs, were purchased from ScienCell Research Laboratories (San Diego). LX-2 was acquired from the American Type Culture Collection (ATCC, Manassas, VA), and primary hHSCs were obtained from the Institute for Liver and Digestive Health, Royal Free Hospital, University College London (London, UK). Cells were cultured accordingly in the growth medium, listed in supplemental Table 1. HHSteCs were passaged when subconfluent in a humidified atmosphere containing 95% air and 5% CO_2_ and were used between passages 3 and 10 for the experiments. Primary hHSCs were isolated from resected liver wedges and were obtained from patients undergoing surgery at the Royal Free Hospital after providing informed consent (EC01.14-RF). Cells were isolated according to a published protocol (44) with modifications for the human liver (45). Upon arrival, a portion of the hHSCs was cultured in SteCM plus 2% FBS with supplement solution. The pCMFLAG-hcJUN plasmid was provided by RIKEN BRC through the National Bio-Resource Project of Ministry of Education, Culture, Sports, Science and Technology (MEXT) (Tsukuba, Ibaraki, Japan). In transient transfection assays, HHSteCs were transfected using Lipofectamine 3000 transfection reagent (Thermo Fisher Scientific, Waltham, MA) for the pCMFLAG-hcJUN vector and Lipofectamine RNAiMAX (Thermo Fisher Scientific) for siRNA transfection.

### Treatment assays

HHSteCs were seeded at a concentration of 1 × 10^5^ cells/ml in SteCM complete medium. The following day, the medium was changed to 2% FBS/SteCM without supplement solution, and cells were stimulated with 4 ng/ml recombinant human basic fibroblast growth factor (FGF2, Wako Pure Chemical Industries, Ltd., Tokyo, Japan) for 72 h, unless otherwise indicated. Recombinant human TGF-β1 (R&D Systems, Minneapolis, MN) diluted in sterile 4 mm HCl containing 1 mg/ml BSA was used as a strong inducer of HSC activation-related genes, αSMA, and collagens. Recombinant human CTGF (46), HGF (47), and PDGF-BB (38) were purchased from PeproTech Inc. (Rocky Hill, NJ). An optimal concentration for each cytokine was determined by using preliminary titrations (supplemental Fig. 4*A*). For the neutralizing assay, anti-FGF2/basic FGF antibodies (2 μg/ml, Millipore, Temecula, CA) were incubated with supplement solution-containing medium for 1 h at 37 °C, and the mixture was added to the cells for 72 h. The effects of the following inhibitors of signaling molecules on CYGB and αSMA expression in HHSteCs were examined at their respective optimal concentrations that were determined from references, and as indicated in Fig. 3*F*: U1026 (MEK inhibitor) (48, 49), triciribine (AKT inhibitor) (50), SB203580 (p38 inhibitor) (51), and SP600125 (JNK inhibitor) (52), all from Wako Pure Chemical Industries, Ltd. Actinomycin D, α-amanitin (53), and G418 (54) (Wako Pure Chemical Industries, Ltd.) were used for transcription and translation inhibition assays. The inhibitors at the indicated concentrations did not show any toxicity on cell viability or proliferation.

### Western blot analyses

Cells were lysed in RIPA buffer (50 mm Tris-HCl, pH 7.5, 150 mm NaCl, 1.0% Nonidet P-40, 0.1% SDS, and 0.5% sodium deoxycholate) containing protease inhibitors (Roche Applied Science, Basel, Switzerland) and phosphatase inhibitors (Thermo Fisher Scientific). The cell lysates were dissolved in SDS sample buffer (50 mm Tris-HCl, pH 6.8, 10% glycerol, 4% SDS, 0.5% bromphenol blue, and 10% β-mercaptoethanol). Aliquots containing 30–40 μg of cellular proteins were separated by 4–10% SDS-PAGE (DRC, Tokyo, Japan) and were transferred to 0.45-μm polyvinylidene difluoride (PVDF) membranes (Bio-Rad). The membranes were incubated overnight at 4 °C with the primary antibodies listed in supplemental Table 2 and were incubated with HRP-conjugated goat anti-mouse or rabbit secondary antibodies (1:5000, Dako, Agilent Technologies, Santa Clara, CA). Proteins were visualized using enhanced chemiluminescence (Thermo Fisher Scientific), and their luminescence was quantified using a luminescent image analyzer, LAS-300 (Fujifilm, Tokyo, Japan). The staining intensity of glyceraldehyde-3-phosphate dehydrogenase (GAPDH) was used as a loading control.

### Quantitative RT-PCR

Cells were lysed in TRIzol reagent (Thermo Fisher Scientific) using the Direct-zol RNA miniPrep kit (Zymo Research, Irvine, CA), and cDNA was generated using SuperScript III reverse transcriptase (Thermo Fisher Scientific) according to the manufacturer's instructions. Quantitative RT-PCR assays were performed using Fast SYBR Green Master Mix (Thermo Fisher Scientific) and an Applied Biosystems 7500 real-time PCR system (Thermo Fisher Scientific) using the primers shown in supplemental Table 3. The relative expression levels were normalized to 18S expression, and fold changes in expression were calculated using the comparative 2^−ΔΔ^*^CT^* method (55).

### ChIP analysis

ChIP assays were carried out using a SimpleCHIP Enzymatic Chromatin IP kit with magnetic beads (Cell Signaling Technology, Danvers, MA) according to the manufacturer's instructions. Briefly, HHSteCs were treated with or without FGF2 (4 ng/ml) for 6 h and were collected with ChIP dilution buffer. Two percent of the supernatant was saved as the input control. Five micrograms of phospho-c-JUN (Ser-73) XP rabbit antibody (Cell Signaling Technology) was added to the diluted chromatin and was incubated overnight. Mock immunoprecipitation was performed in parallel with normal rabbit IgG. Quantitative RT-PCR was performed using ChIP DNA. The data were normalized to that of the input DNA. The primer sequences are shown in supplemental Table 4. The value of the untreated control was set at 1. The results are presented as the relative fold enrichment.

### Immunochemical and phalloidin staining

Cells were fixed with 4% paraformaldehyde (Wako Pure Chemical Industries, Ltd.) and were incubated with polyclonal rabbit anti-CYGB antibody (1:300, in-house) and/or monoclonal mouse anti-human αSMA antibody (1:200, Dako) overnight. Next, cells were washed and stained with Alexa Fluor 594-conjugated goat anti-rabbit and 488-conjugated goat anti-mouse IgG antibodies (1:500, Thermo Fisher Scientific). For F-actin staining, cells were stained with Alexa Fluor 488-conjugated phalloidin (Abcam, Cambridge, UK) for 40 min at room temperature. Cells were counterstained with 4′,6-diamidino-2-phenylindole (DAPI, Dojindo Molecular Technologies, Inc. Tokyo, Japan). The integrated intensity above the threshold of phalloidin-iFluor 488 in one channel was computed and normalized to the number of nuclei (DAPI staining) measured in the other channel, thus giving an average staining intensity per cell using BZ-II analyzer software (Keyence, Osaka, Japan). Formalin-fixed murine liver tissue sections were antigen-retrieved and incubated with anti-αSMA antibody. Images were captured using a BZ-X700-All-in-One fluorescence microscope (Keyence).

### Animal studies

BDL was performed on 6–8-week-old male C57BL/6 mice (Japan SLC, Inc., Shizuoka, Japan). All animal experiments were performed in accordance with the Guide for Animal Experiments, approved by the Animal Research Committee of Osaka City University. Animals were randomly assigned to experimental groups. The surgical procedures were performed under anesthesia via an intraperitoneal injection of 30 mg/kg body weight somnopentyl (Kyoritsu Seiyaku Corp., Tokyo, Japan). Obstructive jaundice was induced by a midline incision in the abdomen and bile duct exposure followed by double ligation with 6-0 silk. Two weeks after surgery, recombinant murine Fgf2 (FGF-basic, PeproTech) at a dose of 60 μg/kg body weight was reconstituted in 100 μl of IMDM and administered via the tail vein twice a week. Animals were euthanized 72 h after the second FGF2 injection. The blood and liver were retrieved for histochemical, biochemical, and molecular analyses. Animals that received an equal volume of IMDM or that underwent sham operation were used as controls (six mice with BDL and three mice without BDL per group). Excised liver specimens were fixed in 10% neutral-buffered formalin and were embedded in paraffin. H&E staining was performed for histological analysis. Sirius red staining (Wako Pure Chemical Industries, Ltd.) for collagen deposition was performed according to the standard procedure. Immunohistochemical analysis on paraffin-embedded sections was performed using a polyclonal rabbit anti-mouse Cygb antibody (1:300, in-house) and a monoclonal mouse anti-αSMA (1:200, DAKO) and stained with Alexa Fluor 594-conjugated goat anti-rabbit and 488-conjugated goat anti-mouse IgG antibodies (1:500, Thermo Fisher Scientific). Nuclei were counterstained with hematoxylin QS (Vector Laboratories, Inc., Burlingame, CA). The percentage areas of Cygb- and αSma-staining HSCs measured in three high-power fields at a magnification of ×10 in five different animals from each group were examined.

### Statistics and reproducibility

All experiments, except for the graphs without error bars, were replicated a minimum of three times. ImageJ analysis was used to determine the optical densities for Western blot analysis and quantitative analysis of Sirius red staining (National Institutes of Health, Bethesda). The level of significance was determined by unpaired *t* test with Welch's correction, one-way ANOVA, or the Mann-Whitney *U* test (repeated measures) for differences across experimental groups and was analyzed with GraphPad Prism6 software. The data are expressed as the mean ± S.D. or median ± S.E. *p* values less than 0.05 were considered to indicate statistical significance.

## Author contributions

M. S. M., T. M., and N. K. designed the experiments and interpreted the results. M. S. M., A. D., Y. O., and L. L. conducted the experiments. K. R. and L. L. provided and characterized the primary human HSCs. J. A. and T. T. performed the MS analysis and acquired the data. M. S. M., T. M., K. R., L. T. T. T., K. I., K. Y., M. P., and N. K. wrote and revised the manuscript.

## Supplementary Material

Supplemental Data

## Footnotes

3 N. T. T. Hai, N. Q. Dat, and N. Kawada, unpublished data.

## Footnotes

2 The abbreviations used are:

ECM: extracellular matrix
αSMA: α-smooth muscle actin
BDL: bile-duct ligation
CLD: chronic liver disease
COLIA1: type I α-1 collagen
COLIA2: type I α-2 collagen
CTGF: connective tissue growth factor
CYGB: cytoglobin
F-actin: filamentous-actin
HGF: hepatocyte growth factor
HHSteC: human hepatic stellate cell line
HSC: hepatic stellate cell
hHSC: human HSC
IMDM: Iscove's modified DMEM
PPARγ: peroxisome proliferator-activated receptor γ
MMP-1: matrix metalloproteinase-1
ROS: reactive oxygen species
SPARC: secreted protein acidic and rich in cysteine
STAP: stellate cell growth supplement solution
SteCM: stellate cell medium
ANOVA: analysis of variance
FGFR: FGF receptor
ALT: aspartate aminotransferase
AST: alanine transaminase.
