# Supplementary material for: Fibroblast growth factor 2 (FGF2) regulates cytoglobin expression and activation of human hepatic stellate cells via JNK signaling
Source: J Biol Chem. 2017 Sep 15;292(46):18961–72. doi: 10.1074/jbc.M117.793794 (PMC5706471; doi:10.1074/jbc.M117.793794)
Supplement: Supplemental Data [file supp_292_46_18961__index.html]

Supplemental Data 

# Fibroblast growth factor 2 (FGF2) regulates cytoglobin expression and activation of human hepatic stellate cells via JNK signaling

## Supplemental Data

- Supplement info.\_revised (.pdf, 547 KB) - Supplement info. revised
