# Supplementary material for: Fibroblast growth factor 2 (FGF2) regulates cytoglobin expression and activation of human hepatic stellate cells via JNK signaling
Source: J Biol Chem. 2017 Sep 15;292(46):18961–72. doi: 10.1074/jbc.M117.793794 (PMC5706471; doi:10.1074/jbc.M117.793794)
Supplement: Supplemental Data [file 10.1074_M117.793794_Supplement_fig_MM.pdf]

SUPPLEMENTAL INFORMATION

METHODS

In-gel Trypsin Digestion and MS/MS Analysis - Supplement was separated by SDS-PAGE on a 4-12% Bis-Tris gel (Thermo Fisher Scientific). After electrophoresis, the gel was stained with Coomassie brilliant blue (Thermo Fisher Scientific). The bands containing intact proteins were excised and washed in 25 mM NH_4HCO_3/50% (v/v) ethanol, followed by 100% ethanol to dehydrate the gel pieces. After removing Coomassie blue, the gel pieces were vacuum-dried, reduced with 10 mM dithiothreitol/50 mM NH_4HCO_3, and were alkylated with 50 mM iodoacetamide/50 mM NH_4HCO_3 for further trypsin digestion. An aliquot of 12.5 ng/µl trypsin in 50 mM NH_4HCO_3 was added to immerse the gel pieces and was incubated at 37°C overnight. Digested peptides were then extracted with repeated rounds of 30% CH_3CN/3% trifluoroacetic acid (TFA). Extracts were vacuum-dried, dissolved in 0.1% TFA and 2 M urea and were analyzed by MS/MS analysis using a LTQ-Orbitrap Velos mass spectrometer (Thermo Fisher Scientific). A database search for the identification of proteins from their MS spectra was conducted using MaxQuant (version 1.3.0.5) supported by the Andromeda search engine for peak detection and quantification. The MS/MS spectra were searched against the UniProt human database with the following search parameters: full tryptic specificity, up to two missed cleavage sites, carbamidomethylation of cysteine residues set as fixed modification, and N-terminal protein acetylation and methionine oxidation as variable modifications. MS/MS tolerance was set at 20 ppm with two or more unique/razor peptides for protein identification and a ratio count of two or more for protein quantification.

D*igital PCR* - Droplet digital PCR (ddPCR; QX200, Bio-rad) was used in this study. Each sample was partitioned into 20,000 droplets, with target and background [reference; AP-3 β-subunit (AP3B1)] DNA randomly distributed among the droplets. Validated following probes were purchased from Bio-Rad: FGFR1 (dHsaCP2500319), FGFR2 (dHsaCP2500320), FGFR3 (dHsaCP1000503) and FGFR4 (dHsaCN803423046) and AP3B1 (dHsaCP2500348). AP3B1 was used as a reference control. The PCR reaction mixture (20 µl) consisted with 10 µl of ddPCR Supermix (no dUTP) for probes, 1 µl of each primer/probe mix (target and reference, labeled with HEX and FAM fluorophores, respectively), and 1-3 µl of the cDNA samples (~100 ng). The emulsified PCR reactions were run in a 96-well plate on a C1000 Touch

Thermal cycler. cDNA was amplified with the following cycle conditions: 95°C for 10 min, followed by 40 cycles of 94°C for 30 s, 60°C for 60 s and 10 min incubation at 98°C. The plate was read on a Bio-Rad QX200 droplet reader using the QuantaSoft v1.4.0 software provided by Bio-rad to assess the number of droplets positive for FGFRs and AP3B1. FGFRs amplification with ddPCR was defined as the FGFRs ratio by calculating FGFRs over AP3B1. Poission equation was used to calculate the concentration based on the number of negative droplets and using the following formula: copies per droplet = -*In*(1-*p)* were *p*= fraction of positive droplet.

SUPPLEMENTAL TABLE 1.

Human HSCs were cultured accordingly in the growth media listed below:

Cells	Company	Growth medium
HHSteCs	ScienCell Research Laboratories (San Diego, CA, USA)	Stellate Cell Medium (SteCM) with 2% fetal bovine serum (FBS), stellate cell growth supplement (Supplement; SteCGS), 100 units of penicillin and 100 µg/ml of streptomycin solution.
LX-2	ATCC (Manassas, VA, USA)	Dulbecco's Modified Eagle's Medium (DMEM, Thermo Fisher Scientific) supplemented with 2% FBS.
Primary hHSC	UCL (London, UK)	Iscove's Modified DMEM (IMDM) supplemented with 20% FBS, 2 mM glutamine, 1× nonessential amino acids, 1.0 mM sodium pyruvate, and 1× antibiotic-antimycotic (all from Thermo Fisher Scientific).

SUPPLEMENTAL TABLE 2.

Primary antibodies used in the western blot analyses

Antibody	Species	Source	Dilution
anti-human CYGB	Rb poly	in house	1:1000
anti-αSMA (Clone 1A4)	Mo mono	Dako	1:3000
anti-FGFR2	Rb poly	Abcam	1:1000
anti-phospho-FGF receptor (Tyr653/654)	Rb poly	CST	1:1000
anti-Raf	Rb poly	SC	1:1000
anti-phospho-c-Raf (Ser259)	Rb poly	CST	1:1000
anti-SEK1/MKK4	Rb poly	CST	1:1000
anti-phospho-SEK1/MKK4 (Ser257/Thr261)	Rb poly	CST	1:1000
anti-SAPK/JNK	Rb poly	CST	1:1000
anti-phospho SAPK/JNK (Thr183/Tyr185)	Rb poly	CST	1:1000
anti-p44/42 MAPK (Erk1/2)	Rb poly	CST	1:1000
anti-phospho p44/42 MAPK (Erk 1/2)	Rb poly	CST	1:1000
anti-Smad3	Rb poly	Abcam	1:1000
anti-phospho Smad3	Rb poly	Abcam	1:1000
anti-AKT	Rb poly	CST	1:1000
anti-phospho AKT (Ser473)	Rb poly	CST	1:1000
anti-c-JUN (60A8)	Rb poly	CST	1:1000
anti-phospho-c-JUN (Ser63) (54B3)	Rb poly	CST	1:1000
anti-GAPDH (clone 6C5)	Mo mono	Millipore	1:10000

Mo mono, mouse monoclonal antibody; Rb poly, rabbit polyclonal antibody. CST: Cell Signaling Technology, SC: Santa Cruz

SUPPLEMENTAL TABLE 3.

List of primers used for quantitative RT-PCR in this study

Gene	Forward (5'-3')	Reverse (5'-3')
hαSMA	CAGCCAAGCACTGTCAGG	CCAGAGCCATTGTCACACAC
hCYGB	CGAGATGGAGATCGAGCG	CGAGGGGAAGTTCACAAAGA
hPPARγ	AGGCCATTTTCTCAAACGAG	GAGAGATCCACGGAGCTGAT
hCOLIA1	AAGAGGAAGGCCAAGTCGAG	CACACGTCTCGGTCATGGTA
hCOLIA2	GAAAAGGAGTTGGACTTGGC	AGCAGGTCCTTGGAAACCTT
hSPARC	CTTCAGACTGCCCGGAGA	GAAAGAAGATCCAGGCCCTC
hDESMIN	GAAGCTGCTGGAGGGAGAG	ATGGACCTCAGAACCCCTTT
hNTF3	GAAACGCGATGTAAGGAAGC	GGTTTGGGATGTTTTGCACT
hRBP1	TTGAGGAGGATCTGACAGGC	TCCTTCTCACCCTTCTGCAC
hLRAT	TACTGCAGATATGGCACCCC	CCAAGACTGCTGAAGCAAGA
hcJUN	CCCCAAGATCCTGAAACAGA	CCGTTGCTGGACTGGATTAT
h18S	AGTCCCTGCCCTTTGTACACA	CGATCCGAGGGCCTCACTA
mCygb	GCTGTATGCCAACTGCGAG	CCTCCATGTGTCTAAACTGGC
mαSma	ACTGGGACGACATGGAAAAG	GTTCAGTGGTGCCTCTGTCA
mColIa1	ACATGTTCAGCTTTGTGGACC	TAGGCCATTGTGTATGCAGC
m18s	CGGCTACCACATCCAAGGAA	ATTGGAGCTGGAATTACCGC

Note: h or m indicates primers for human or mouse, respectively.

SUPPLEMENTAL TABLE 4.

List of primers used for ChIP-quantitative RT-PCR in this study

Gene	Forward (5'-3')	Reverse (5'-3')
Primer 1	TCCAACACTCCCGAGCTTTC	GAGGGGCTCCTGTTCGC
Primer 2	ATCTAATGCCTCCTGGGGGT	GGGAGTGGTGTGAGAACCTG

SUPPLEMENTAL FIGURE S1. (A) Expression of HSC-associated genes in HHSteCs compared with that in LX-2 cells. Expression of HSC-associated genes *DESMIN*, *NTF3*, *RBP1* and *LRAT* in HHSteCs compared with that in LX-2 cells. The data were normalized to *18S* mRNA, and error bars indicate SD. *NTF3*: neurotrophin-3; *RBP1*: retinol-binding protein-1; *LRAT*: lecithin retinol acyltransferase. The data are expressed as the mean ± SD from three independent experiments. $*p < 0.05$ using unpaired t-test with Welch's correction.

SUPPLEMENTAL FIGURE S2. (A) MS/MS analysis of the identification of human FGF2 in Supplement. MS/MS spectra for the parent ion 536.2. The amino acid sequence AILFLPMSAK for FGF2 was confirmed by analyzing b- and y-ions derived from the peptide ion.

SUPPLEMENTAL FIGURE S3. FGF2 is identified as a CYGB inducer. (A) Phase-contrast images showing Supplement-induced morphological changes and recovery by 2 h of pre-incubation with anti-FGF2 antibody (2 µg/ml) in HHSteCs. Bar, 100 µm. (B) Dose-dependent effect of anti-FGF2 antibody (0, 0.5, 1, 2, and 4 µg/ml) on the CYGB and αSMA protein levels in Supplement-treated HHSteCs. (C) Copy number of *FGFR1*, *FGFR2*, *FGFR3* and *FGFR4* were quantified in triplicate in HHSteCs stimulated with FGF2 (4 ng/ml) for 3 days. cDNA was synthesized from 100 ng of isolated RNA by reverse transcription and template cDNA (1 µl for *FGFR1* and *FGFR3*, 2 µl for *FGFR4* and 3 µl for *FGFR2* probes) was used for ddPCR. Samples were partitioned using the Droplet Generator and thermal cycled to end-point. PCR reaction was read using Droplet Reader and results were analyzed using QauantaSoft (Bio-Rad). The average concentration of FGFRs is represented in copy number calculated using Poisson equation. Error bars indicate SD with $*p < 0.05$ using unpaired ttest. (D) The graph shows the mRNA expression of *COLIA1* in HHSteCs with untreated control (closed circles), FGF2 (4 ng/ml, open triangles), and Supplement (1×, open squares); error bars indicate SD.

SUPPLEMENTAL FIGURE S4. (A) Dose dependency of recombinant CTGF, HGF and PDGF on CYGB expression in HHSteCs. GAPDH were used as a loading control.

Figure S1.

A

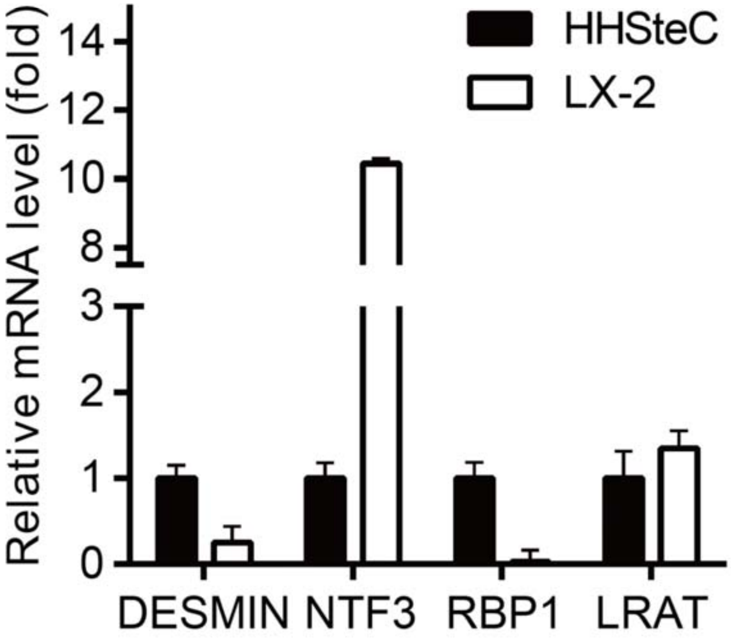

HHSteC
LX-2
Relative mRNA level (fold)
14
12
10
8
3
2
1
0
DESMIN
NTF3
RBP1
LRAT

Figure S2.

A

Accession	Name	m/z	Sequence
A0A087WUF6	Fibroblast growth factor 2 [_HUMAN]	553.81744	AILFLPMSAK

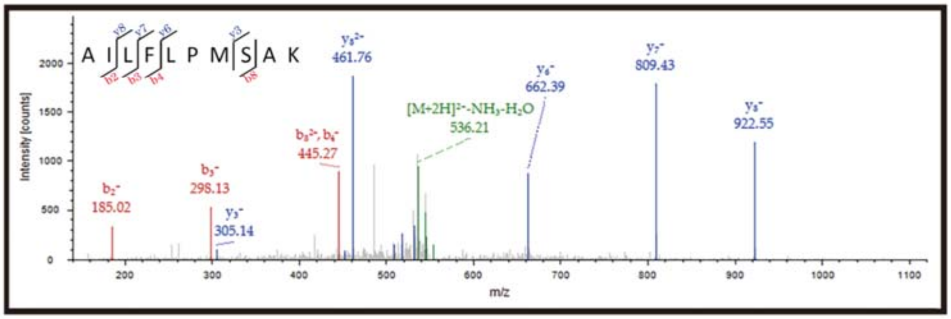

FIGURE S3.

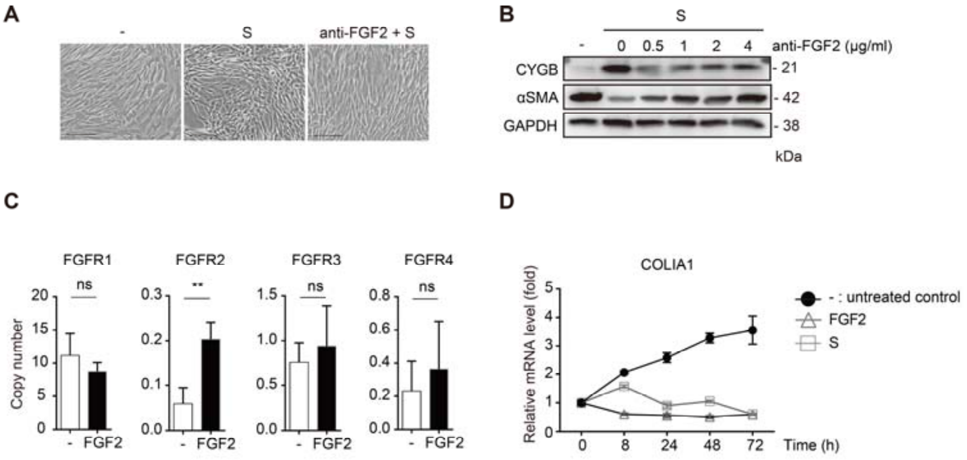
A
-
S
anti-FGF2 + S
B
S
-
0
0.5
1
2
4
anti-FGF2 (μg/ml)
CYGB
αSMA
GAPDH
- 21
- 42
- 38
kDa
C
FGFR1
FGFR2
FGFR3
FGFR4
ns
**
ns
ns
Copy number
-
FGF2
D
COLIA1
Relative mRNA level (fold)
- : untreated control
FGF2
S
Time (h)
0
8
24
48
72

FIGURE S4.

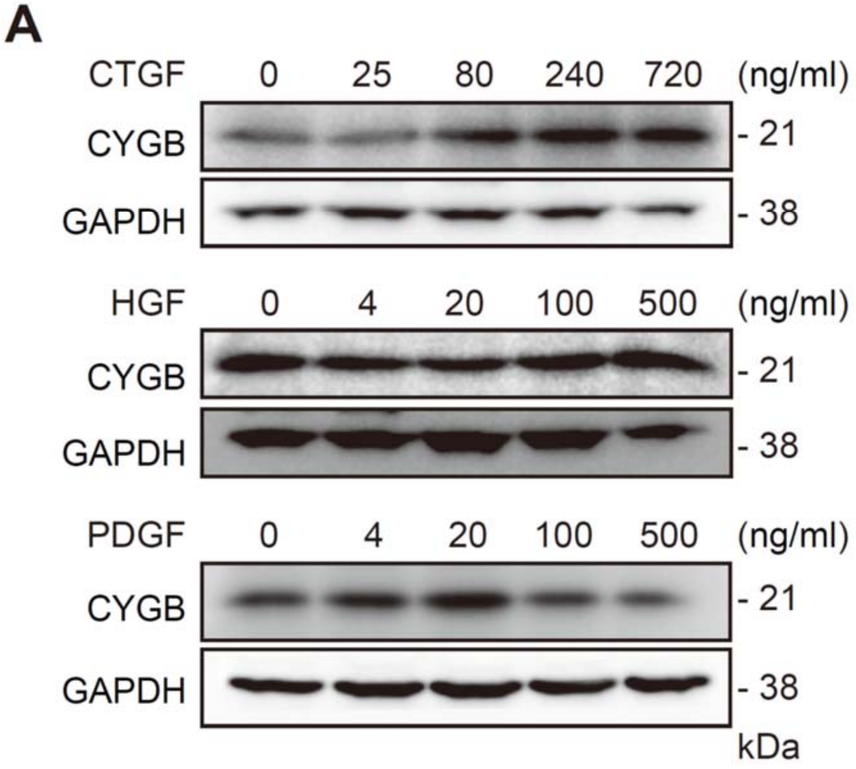
A
CTGF 0 25 80 240 720 (ng/ml)
CYGB - 21
GAPDH - 38
HGF 0 4 20 100 500 (ng/ml)
CYGB - 21
GAPDH - 38
PDGF 0 4 20 100 500 (ng/ml)
CYGB - 21
GAPDH - 38
kDa
